# Suppressive effects of (-)-tubaic acid on RANKL-induced osteoclast differentiation and bone resorption

**DOI:** 10.1080/19768354.2023.2166107

**Published:** 2023-01-12

**Authors:** Soomin Lim, Hye Jung Ihn, Ju Ang Kim, Jong-Sup Bae, Jung-Eun Kim, Yong Chul Bae, Hong-In Shin, Tae Hoon Kim, Eui Kyun Park

**Affiliations:** a Department of Oral Pathology and Regenerative Medicine, School of Dentistry, IHBR, Kyungpook National University, Daegu, Republic of Korea; b Cell and Matrix Research Institute (CMRI), Kyungpook National University, Daegu, Republic of Korea; c College of Pharmacy, CMRI, Research Institute of Pharmaceutical Sciences, Kyungpook National University, Daegu, Republic of Korea; d Department of Molecular Medicine, CMRI, School of Medicine, Kyungpook National University, Daegu, Republic of Korea; e Department of Oral Anatomy and Neurobiology, School of Dentistry, Kyungpook National University, Daegu, Republic of Korea; f Department of Food Science and Biotechnology, Daegu University, Gyeongsan, Republic of Korea

**Keywords:** (-)-tubaic acid, osteoclast, bone resorption, nuclear factor of activated T-cells cytoplasmic 1 (NFATc1)

## Abstract

Regulation of osteoclastogenesis and bone-resorbing activity can be an efficacious strategy for treating bone loss diseases because excessive osteoclastic bone resorption leads to the development of such diseases. Here, we investigated the role of (-)-tubaic acid, a thermal degradation product of rotenone, in osteoclast formation and function in an attempt to identify alternative natural compounds. (-)-Tubaic acid significantly inhibited receptor activator of nuclear factor-κB ligand (RANKL)-mediated osteoclast differentiation at both the early and late stages, suggesting that (-)-tubaic acid affects the commitment and differentiation of osteoclast progenitors as well as the cell-cell fusion of mononuclear osteoclasts. (-)-Tubaic acid attenuated the activation of extracellular signal-regulated kinase (ERK) and expression of nuclear factor of activated T-cells cytoplasmic 1 (NFATc1) and its target genes in response to RANKL. Furthermore, a pit-formation assay revealed that (-)-tubaic acid significantly impaired the bone-resorbing activity of osteoclasts. Our results demonstrated that (-)-tubaic acid exhibits anti-osteoclastogenic and anti-resorptive effects, indicating its therapeutic potential in the management of osteoclast-related bone diseases.

## Introduction

Osteoclastic bone resorption and osteoblastic bone formation are balanced in physiological states that sustain bone mass and mineral homeostasis (Siddiqui and Partridge 2016). However, bone-resorbing activity increases with aging and pathological conditions, and the imbalance in bone remodeling ultimately reduces the amount and quality of bones (Almeida and O'Brien 2013). Several skeletal diseases are strongly associated with enhanced osteoclast activation; therefore, osteoclasts have been considered promising targets for the development of therapeutic interventions to manage osteoclast-related bone diseases.

Typically, osteoclasts are derived from monocyte/macrophage progenitors through several steps. The differentiation process is dependent on macrophage colony-stimulating factor (M-CSF) supporting the proliferation and survival of osteoclast precursors and receptor activator of nuclear factor-κB ligand (RANKL) promoting their differentiation into osteoclasts (Cappellen et al. 2002). The interaction between RANKL and its receptor RANK, expressed on osteoclast precursors, acts as a stimulating signal and activates multiple signal transduction pathways, including mitogen-activated protein kinases (MAPKs) and nuclear factor-κB (NF-κB). These ultimately stimulate the induction of the essential transcription factor, the nuclear factor of activated T-cells cytoplasmic 1 (NFATc1), which regulates the expression of genes required for osteoclast differentiation and function (Takayanagi et al. 2002; Park et al. 2017).

**Figure 1.**
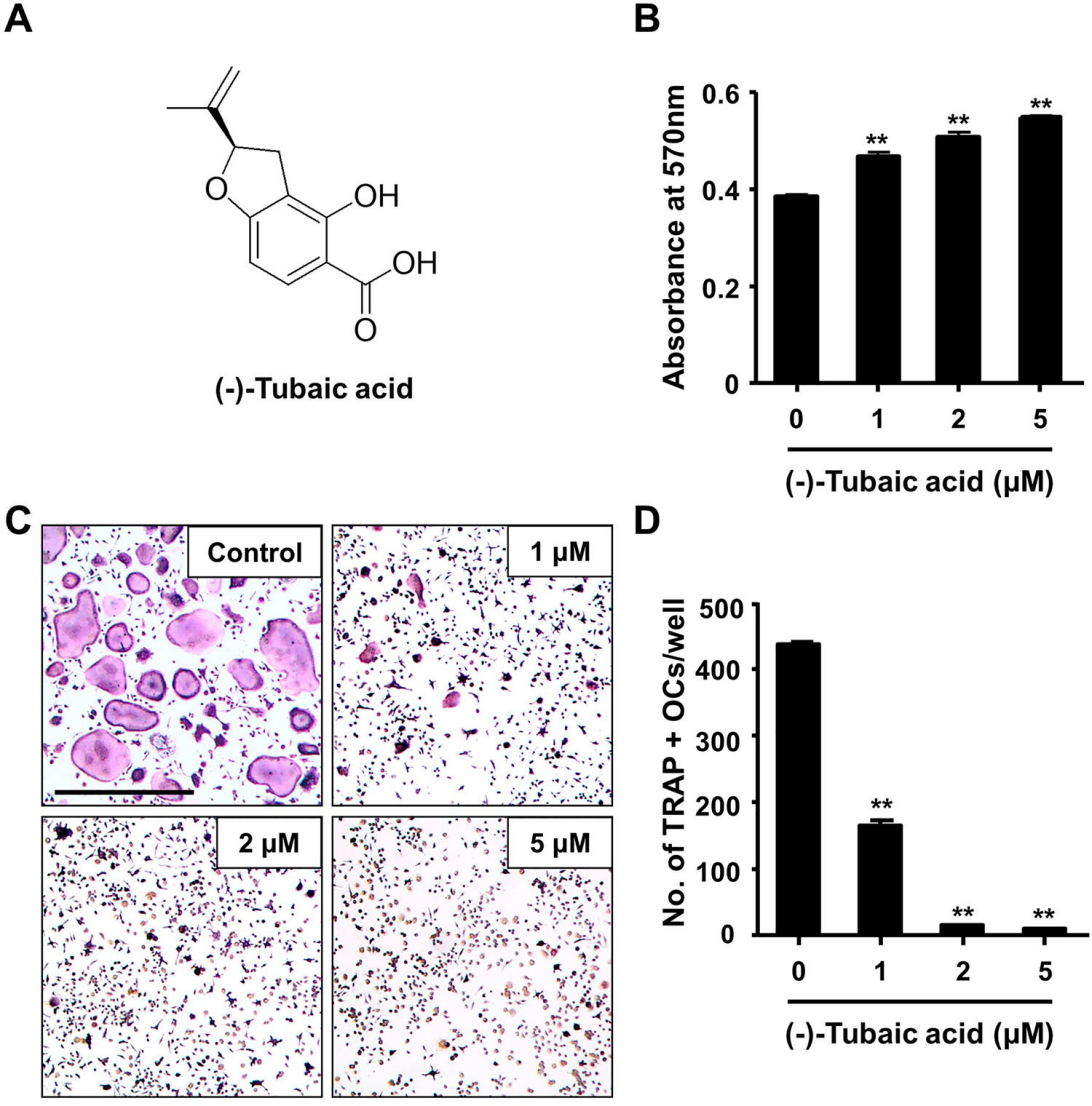
(-)-Tubaic acid suppresses RANKL-mediated osteoclastogenesis without inducing cytotoxicity. (A) Chemical structure of (-)-tubaic acid. (B) Bone marrow-derived macrophages (BMMs) were incubated with M-CSF (10 ng/ml) and various doses of (-)-tubaic acid, and cell viability was assessed using the MTT assay. (C) The BMMs were cultured with M-CSF (10 ng/ml) and RANKL (20 ng/ml) in the presence of (-)-tubaic acid or vehicle. After four days, the cells were stained to assess TRAP activity. Scale bar, 50 μm. (D) Quantification of TRAP-positive multinucleated cells (MNCs) with over three nuclei. **p < 0.01 versus vehicle-treated control, *t*-test.

Rotenoid compounds exhibit diverse biological and pharmacological functions, including antibacterial, antifungal, anticancer, and anti-inflammatory functions (Fang and Casida 1998; Takashima et al. 2002; Mathias et al. 2005). Rotenone is a member of the rotenoid family, and attenuates osteoclast differentiation and suppresses inflammatory bone loss (Kwak et al. 2010). Rotenone derivative, 1′,2′-dihydrorotenone, also exerts a negative activity on osteoclastogenesis (Lee et al. 2011). One of degradation products of rotenone is a dihydrobenzofuran (coumaran) derivative, tubaic acid (Figure 1(a), Fig. S1) (Cheng et al. 1972). Dihydrobenzofuran is proposed as an important heterocyclic motif in biologically active natural products (Choi et al. 2015; Sunden et al. 2016). Numerous natural bioactive compounds carrying a 2,3-dihydrobenzofuran scaffold play beneficial roles in inflammation and HIV infection. Lithospermic acid, containing the 2,3-dihydrobenzofuran skeleton isolated from *Salvia miltiorrhiza* roots, exhibits anti-HIV activity in H9 cells (Abd-Elazem et al. 2002). Inagaki et al. reported that the 2,3-dihydrobenzofuran derivative, (E)-5-(7-tert-Butyl-3,3-dimethyl-2,3-dihydrobenzofuran-5-ylmethylene)-2-ethyl-1,2-isothiazolidine 1,1-dioxide, inhibited PGE2 production and demonstrated anti-inflammatory activity in a carrageenan-induced footpad edema model (Inagaki et al. 2003). Furthermore, kadsurenone, a natural platelet-activating factor (PAF) antagonist, has a 2,3-dihydrobenzofuran structure and attenuates breast cancer (BC) cell-induced osteoclast formation as well as PAF-induced BC cell migration, supporting it as a potential therapeutic agent for BC-induced bone metastases (Hou et al. 2018). Although tubaic acid is a pivotal intermediate in rotenone synthesis and contains a 2,3-dihydrobenzofuran skeleton, little is known about its biological functions. Thus, we examined the therapeutic potential of tubaic acid in osteoclast-related bone diseases in this study. Furthermore, we investigated the effects of tubaic acid on osteoclastogenesis, bone resorption, and related action mechanisms.

## Materials and methods

Fetal bovine serum (FBS) and α-minimum essential medium (α-MEM) were obtained from Gibco BRL (Grand Island, NY, USA). Recombinant murine M-CSF and RANKL were purchased from R&D Systems (Minneapolis, MN, USA). The acid phosphatase, leukocyte (tartrate-resistant acid phosphatase: TRAP) kit, methylthiazolyldiphenyl-tetrazolium bromide (MTT), dimethyl sulfoxide (DMSO), and all other reagents were purchased from Sigma–Aldrich (St. Louis, MO, USA). (-)-Tubaic acid (4-hydroxy-2-(1-methylethenyl)-2,3-dihydrobenzofuran-5-carboxylic acid) was isolated by the thermal transformation of rotenone, and the isolation process is described later in this section.

### Thermal transformation of rotenone and isolation of (-)-tubaic acid

A sample solution of rotenone (1.0 g) in H_2_O (2.0 L) in capped vials was autoclaved at 121°C for 12 h. The dried reactant was directly subjected to column chromatography over a YMC GEL ODS AQ 120-50S column (1.1 cm i.d. × 36 cm) with aqueous MeOH to yield pure (-)-tubaic aid (16.6 mg). High-performance liquid chromatography (HPLC) analysis was performed on a YMC-Pack ODS A-302 column (4.6 mm i.d. × 150 mm; YMC Co., Ltd.), which consisted of a linear gradient that started with 10% (v/v) MeCN in 0.1% HCOOH/H_2_O (detection: UV 280 nm; flow rate: 1.0 ml/min; at 40°C), increased to 80% MeCN over 23 min, and then to 100% MeCN over 5 min. The structure of the newly generated compound was determined based on the interpretation of the spectroscopic data.

### Osteoclast differentiation and TRAP staining

Osteoclasts were generated from bone marrow-derived macrophages (BMMs) as previously described (Ihn et al. 2019; Ihn et al. 2019; Ahn et al. 2021). Bone marrow cells obtained from 6–8-week-old C57BL/6 mice (Dae Han Bio Link, Chungbuk, Korea) were incubated in α-MEM containing 10% FBS for 24 h, and nonadherent cells were incubated in the presence of 30 ng/ml M-CSF for three days to generate BMMs. To induce osteoclastogenesis, BMMs were cultured with RANKL (20 ng/ml) and M-CSF (10 ng/ml) in the presence of (-)-tubaic acid (0, 1, 2, or 5 μM). After four days, the cultured cells were stained using the TRAP staining kit, and TRAP-positive cells containing over three nuclei were quantified by manually counting under a Leica light microscope (Leica, Germany).

### Cytotoxicity assay

The cytotoxic effects were evaluated using the MTT assay (Jeong et al. 2021). BMMs were incubated with different doses of (-)-tubaic acid in the presence of M-CSF (10 ng/ml). After three days, the MTT reagent was added to each well, and the cells were incubated at 37°C for 2 h. The formazan crystals were solubilized with DMSO, and cell viability was assessed by measuring the absorbance at 570 nm using a 96-well microplate reader (Bio-Rad, Hercules, CA, USA).

### Real-time PCR

Total RNA was extracted using TRI-solution (Bio Science Technology, Daegu, Korea), according to the manufacturer’s instructions, and complementary DNA (cDNA) was synthesized using SuperScript II Reverse Transcriptase (Invitrogen, Carlsbad, CA, USA). Quantitative real-time PCR was performed using a LightCycler 1.5 real-time PCR system (Roche Diagnostics, Basel, Switzerland) and SYBR Premix Ex Taq (Takara Bio Inc., Shiga, Japan). Primer sequences for osteoclast-specific genes: TRAP (*Acp5*), 5′-TCCCCAATGCCCCATTC-3′ and 5′-CGGTTCTGGCGATCTCTTTG-3′; *Ctsk*, 5′-GGCTGTGGAGGCGGCTAT-3′ and 5′-AGAGTCAATGCCTCCGTTCTG-3′; *Dcstamp*, 5′-CTTCCGTGGGCCAGAAGTT-3′ and 5′-AGGCCAGTGCTGACTAGGATGA-3′; *Nfatc1*, 5′-ACCACCTTTCCGCAACCA-3′ and 5′-TTCCGTTTCCCGTTGCA-3′.

### Immunofluorescence staining

BMMs were plated on glass coverslips and incubated in α-MEM containing 10% FBS, 10 ng/ml M-CSF, and 20 ng/ml RANKL, with or without 5 μM (-)-tubaic acid. After four days, the cells were fixed with 4% paraformaldehyde, treated with 0.25% Triton X-100, and stained with an anti-NFATc1 antibody (Santa Cruz Biotechnology, Santa Cruz, CA, USA), followed by an Alexa Fluor-488-conjugated secondary antibody. Rhodamine-conjugated phalloidin (Cytoskeleton, Denver, CO, USA) and 4′,6-diamidino-2-phenylindole dihydrochloride (DAPI; Santa Cruz Biotechnology, Santa Cruz, CA, USA) were used to stain F-actin and cell nuclei, respectively. Cells with actin rings or nuclear NFATc1 were counted from 10 random selected views using the Image J software (NIH, Bethesda, MA, USA).

### Western blotting

Cells were lysed in RIPA lysis buffer supplemented with protease and phosphatase inhibitors, and the total protein concentration was measured using a BCA Protein Assay Kit (Pierce Biotechnology, Rockford, IL, USA). Proteins (30 μg) were subjected to 10% SDS-PAGE and transferred onto a nitrocellulose membrane (Whatman, Florham Park, NJ, USA). Membranes were immersed in 3% non-fat milk in Tris-buffered saline with 0.1% Tween 20 (TBS-T) for 1 h before incubation with primary antibodies against p-p38, p-JNK, p-ERK, p-MEK, p-AKT, p-IκBα (Cell Signaling Technology, Danvers, MA, USA), and β-actin (Sigma–Aldrich, St. Louis, MO, USA) at 4°C. Immunoreactive bands were detected using a WesternBright ECL kit (Advansta, Menlo Park, CA, USA) and recorded using a chemiluminescence imager (Azure Biosystems, Inc., Dublin, CA, USA).

### *In vitro* resorption assay

BMMs seeded on bone slices (IDS Nordic, Herlev, Denmark) were incubated in an osteoclastogenic medium for three days, followed by treatment with vehicle or 5 μM (-)-tubaic acid. After two days, the bone slices were washed and immersed in hematoxylin solution to visualize the resorption pits. The area of the pits was quantified by analyzing 10 randomly selected images using i-Solution image analysis software (IMT i-Solution, Daejeon, Korea).

### Statistical analysis

Experiments were conducted in triplicate and repeated three times, and the results are presented as the mean ± standard deviation (SD). A two-tailed student’s *t*-test was used to determine statistical significance. *P < 0.05 or **P < 0.01 was considered statistically significant.

## Results

### (-)-Tubaic acid suppresses RANKL-mediated osteoclast differentiation in BMMs

**Figure 2.**
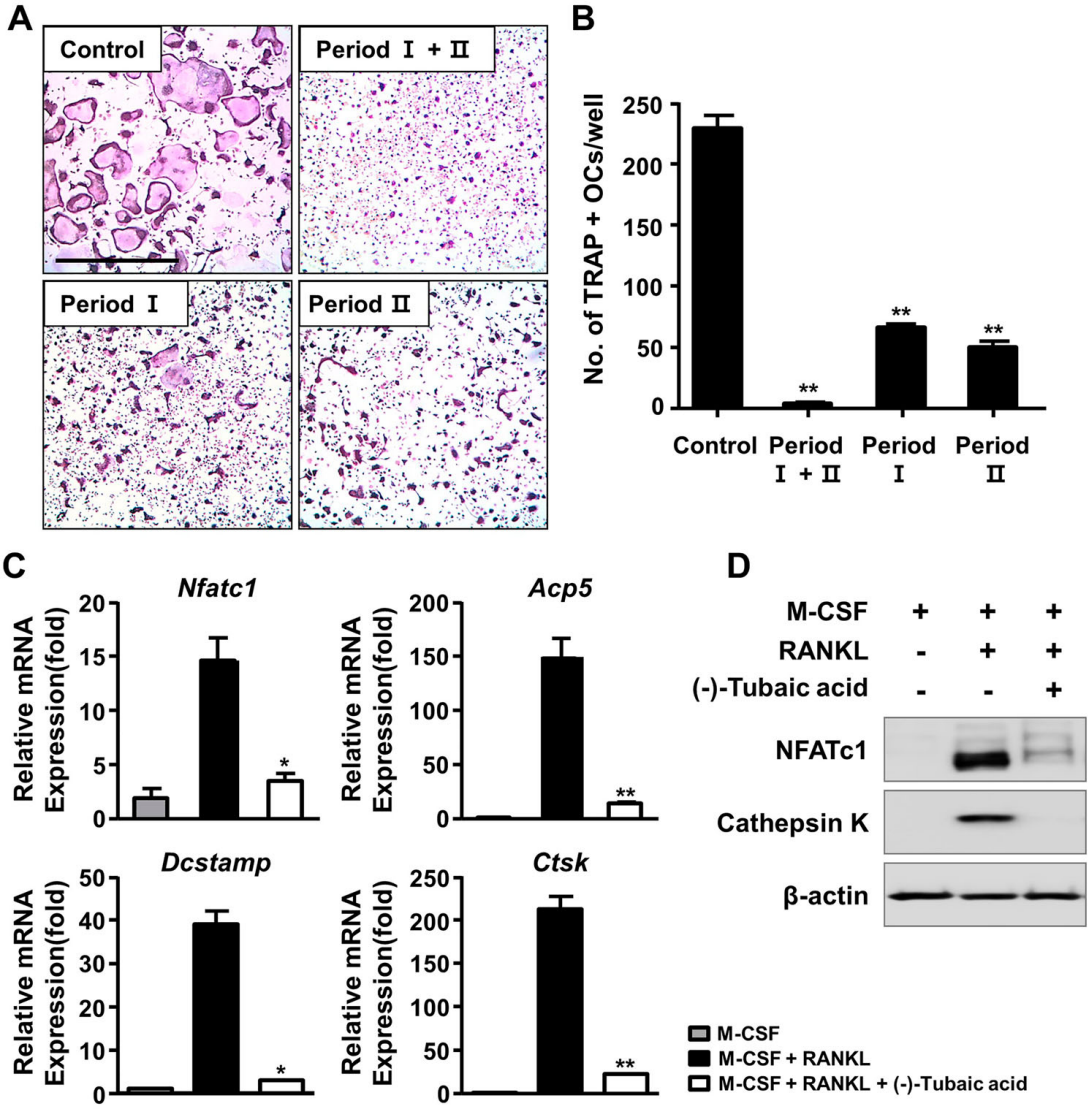
(-)-Tubaic acid inhibits early and late stages of osteoclast differentiation and osteoclast-specific marker expression. (A) The BMMs were cultured in the presence of M-CSF (10 ng/ml) and RANKL (20 ng/ml), and the cells were treated with (-)-tubaic acid (5 μM) for the indicated period. Period I : from days 0–2, Period II : from days 2–4. Scale bar, 50 μm. (B) Quantification of TRAP-positive MNCs. (C, D) The BMMs were incubated in the presence of M-CSF (10 ng/ml) and RANKL (20 ng/ml) with or without (-)-tubaic acid (5 μM) for four days. The mRNA (C) and protein (D) expression levels of osteoclast markers were assessed by real-time PCR and immunoblotting, respectively. *p < 0.05, **p < 0.01 versus vehicle-treated control, *t*-test.

To explore the effects of (-)-tubaic acid (Figure 1(a)) on RANKL-induced osteoclastogenesis, we first investigated whether (-)-tubaic acid affects the viability of BMMs. The MTT assay results revealed that (-)-tubaic acid did not exhibit cytotoxicity, even at 5 μM, and increased the viability of BMMs (Figure 1(b)). When BMMs were cultured in an osteoclastogenic medium with different doses of (-)-tubaic acid for four days, (-)-tubaic acid treatment significantly suppressed the formation of TRAP-positive multinucleated cells (MNCs) from BMMs in a dose-dependent manner compared with that in the vehicle-treated control (Figure 1(c)). Notably, 5 μM (-)-tubaic acid almost completely blocked osteoclast formation (97.79% inhibition; Figure 1(d)). To further confirm the stage at which (-)-tubaic acid impaired osteoclast formation, (-)-tubaic acid (5 μM) was added to the culture medium at different periods, as previously reported (Ihn et al. 2017; Lim et al. 2020). Addition of (-)-tubaic acid in the early (Period I: from days 0–2) or late (Period II: from days 2–4) stages of osteoclast differentiation, the formation of TRAP-positive MNCs was significantly decreased (Figure 2(a)). The number of MNCs was reduced by 71.2% and 78.2% in the early and late treatments, respectively (Figure 2(b)), indicating that (-)-tubaic acid can affect both stages, including the commitment to osteoclastic differentiation and cell-cell fusion.

### (-)-Tubaic acid reduces osteoclast marker expression

**Figure 3.**
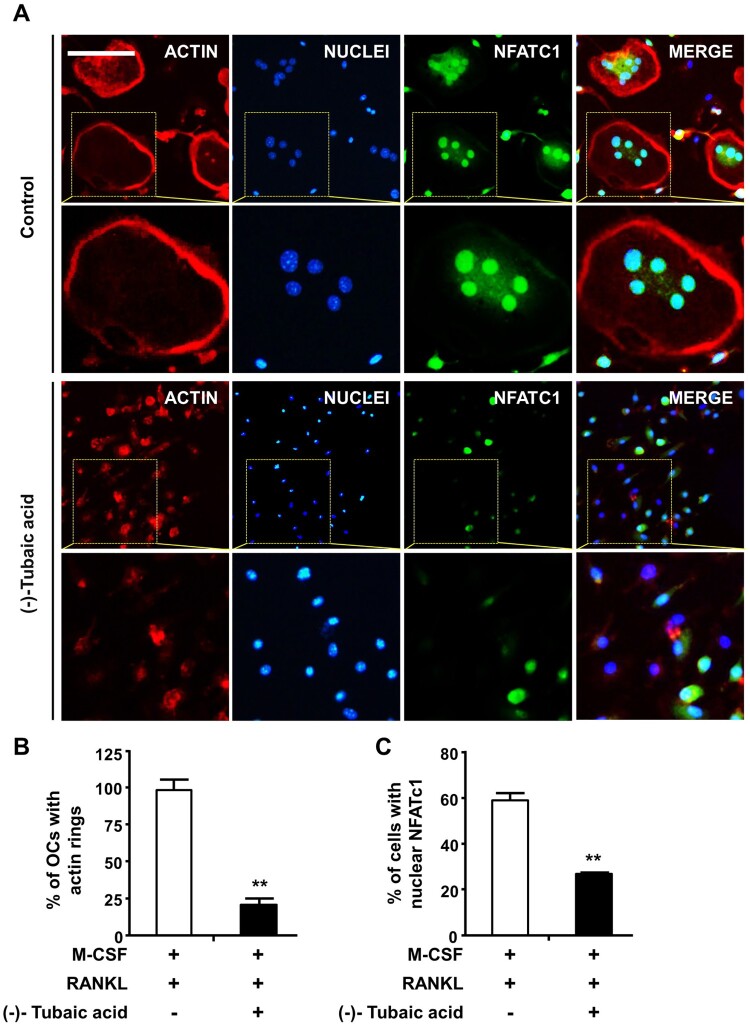
(-)-Tubaic acid impairs actin ring formation and decreases nuclear localization of NFATc1. (A) BMMs were incubated on glass coverslips in an osteoclastogenic medium containing M-CSF (10 ng/ml) and RANKL (20 ng/ml) with (-)-tubaic acid (5 μM) or vehicle. After four days, the cells were fixed and probed with anti-NFATc1 antibody (green), followed by staining with rhodamine-conjugated phalloidin (red) and DAPI (blue). Yellow dashed rectangles were magnified in the lower panels. Scale bar, 50 μm. Quantification of the percentages of (B) cells displaying actin rings and (C) cells with nuclear NFATc1. **p < 0.01 versus vehicle-treated control, *t*-test.

To further determine the anti-osteoclastogenic potential of (-)-tubaic acid, mRNA expression levels of NFATc1 and its target genes related to osteoclast differentiation and function were assessed. As shown in Figure 2(c), (-)-tubaic acid (5 μM) suppressed the expression of *Nfatc1* and downstream osteoclast marker genes, such as TRAP (*Acp5*), *Dcstamp*, and cathepsin K (*Ctsk*). The protein levels of NFATc1 and cathepsin K were significantly down-regulated by (-)-tubaic acid treatment (Figure 2(d)). We also observed reduced nuclear localization of NFATc1 in (-)-tubaic acid-treated cells (Figure 3(a,c)).

### (-)-Tubaic acid suppresses the formation of actin rings and resorption pits

**Figure 4.**
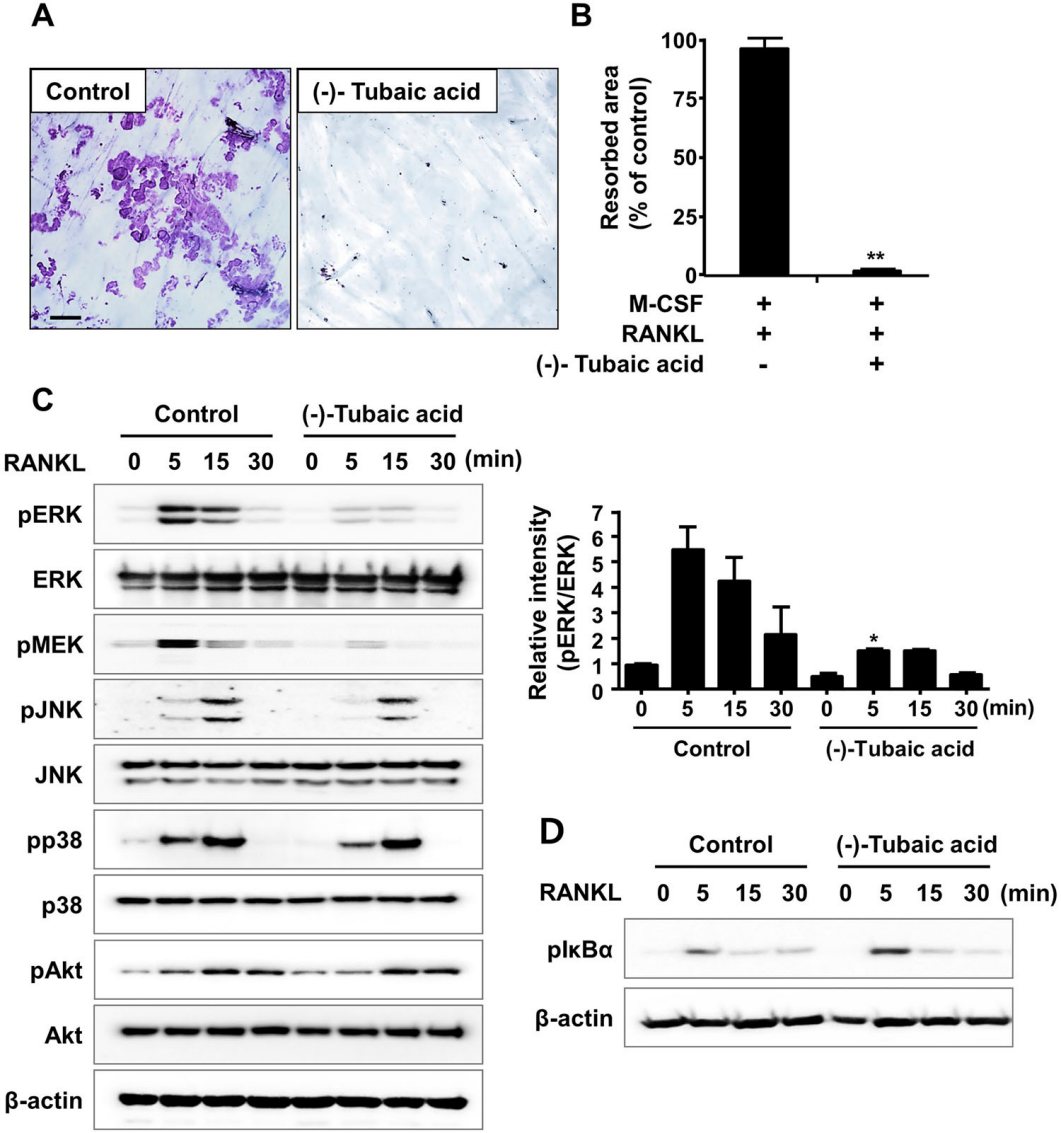
(-)-Tubaic acid reduces resorption pit formation and attenuates RANKL-induced activation of ERK. (A) The BMMs were cultured on bone slices with M-CSF (10 ng/ml) and RANKL (20 ng/ml) to induce osteoclast differentiation. After three days, the cells were treated with (-)-tubaic acid (5 μM) or vehicle for an additional two days. The bone slices were stained with hematoxylin. Scale bar, 100 μm. (B) Quantification of the resorbed area. (C, D) Serum-starved BMMs were pretreated with (-)-tubaic acid (5 μM) or vehicle for 1 h, followed by stimulation with 50 ng/ml RANKL for indicated times. The phosphorylation levels of (C) ERK, Akt, JNK, p38, and (D) IκBα were examined by western blotting. The graph (right panel) represents the relative band intensity of phosphorylated ERK. *p < 0.05, **p < 0.01, *t*-test.

As osteoclasts form actin rings around the cell periphery, which serves as an indicator of active osteoclasts and are vital for bone resorption (Boyce et al. 1992), the effects of (-)-tubaic acid on actin ring formation and bone resorption activity were examined. Phalloidin staining revealed that F-actin rings were present in the vehicle-treated control group, whereas the addition of 5 μM (-)-tubaic acid impaired actin ring formation (Figure 3(a,b)). Next, we performed a pit formation assay to explore the effects of (-)-tubaic acid on osteoclastic bone resorption. BMMs were cultured on bone slices in an osteoclast induction medium for three days, and the cells were treated with either 5 μM (-)-tubaic acid or vehicle. Numerous hematoxylin-stained resorption pits were formed in the vehicle-treated control; however, (-)-tubaic acid treatment led to a significant decrease in the resorbed area (Figure 4(a,b)).

### (-)-Tubaic acid attenuates RANKL-mediated ERK activation

Mitogen-activated protein kinases [c-Jun-N-terminal kinase (JNK), extracellular signal-regulated kinase (ERK), and p38] and NF-κB pathways are activated in response to RANKL stimulation, and they are essential for osteoclast differentiation and function (Mizukami et al. 2002). As (-)-tubaic acid exhibits anti-osteoclastogenic and anti-resorptive potential, its effect on RANKL-mediated signaling pathways was investigated to better understand the molecular mechanism. After pretreatment with (-)-tubaic acid or vehicle, BMMs were stimulated with RANKL, and the phosphorylation levels of signaling molecules were assessed by immunoblotting. Upon RANKL treatment, phosphorylation of MAPKs and IκBα increased within 15 min in control cells (Figure 4(c,d)). In contrast, pretreatment with (-)-tubaic acid reduced the RANKL-induced phosphorylation of MEK, the upstream activator of ERK, and ERK without affecting the phosphorylation of JNK, p38, Akt, and IκBα (Figure 4(c,d)).

## Discussion

Natural compounds and their derivatives have attracted attention as essential resources for the development of therapeutic agents for decades, owing to their potential medicinal properties. Phytoestrogens, including isoflavones, lignans, and coumestrol, are polyphenolic compounds, and several reports highlight their beneficial effects on bone health (Castelo-Branco and Cancelo Hidalgo 2011; Abdi et al. 2016). Tubaic acid is produced by the degradation of rotenone, a naturally occurring isoflavone compound that exhibits antimicrobial activity (Obara et al. 1976). Tubaic acid contains a 2,3-dihydrobenzofuran scaffold, which is considered a vital element in many biologically active natural compounds (Qin et al. 2017). To date, the biological and pharmacological properties of tubaic acid have rarely been studied. In this study, we demonstrated that (-)-tubaic acid inhibited RANKL-induced osteoclastogenesis and suppressed bone-resorbing activity.

M-CSF plays essential roles in the survival, proliferation, and differentiation of osteoclast precursor cells, and binding of M-CSF to its receptor, c-Fms, activates various signaling molecules, including ERK and PI3 K/Akt (Richardson et al. 2015). BMMs are widely used as osteoclast precursors in an *in vitro* osteoclastogenesis system, and an increase in cell viability was observed when BMMs were treated with (-)-tubaic acid (Figure 1(b)), suggesting that tubaic acid may affect one of the c-Fms signaling pathways to induce cell survival or proliferation. Osteoclast differentiation comprises several steps, including commitment and differentiation to mononuclear preosteoclasts, cellular fusion to multinuclear osteoclasts, and activation of bone-resorbing osteoclasts (Xing et al. 2012). (-)-Tubaic acid significantly suppressed both the early and late stages of osteoclast differentiation, with over 70% inhibition of osteoclast formation (Figure 2(b)), indicating that it impairs preosteoclast formation and cellular fusion in the early and late stages, respectively. We also confirmed the inhibitory effects of (-)-tubaic acid at the molecular level by assessing changes in the expression of osteoclast-specific markers. The expression of NFATc1 and osteoclast-related genes was attenuated in the presence of (-)-tubaic acid (Figure 2(c,d)), which is consistent with the TRAP staining results, indicating that (-)-tubaic acid has anti-osteoclastogenic properties. Cell-cell fusion required for the formation of multinucleated cells during osteoclastogenesis is crucial for efficient bone resorption (Lee et al. 2009), and DC-STAMP is well established as an essential regulator of this process (Yagi et al. 2005). Genetic ablation of *Dcstamp* results in the failure to generate multinuclear osteoclasts, leading to a reduction in bone-resorbing activity (Yagi et al. 2005). Although mononuclear osteoclastic cells in DC-STAMP knockout mice can resorb bone, their resorbing activity is lower than that of multinucleated osteoclasts (Yagi et al. 2005). Osteoclastic resorption is also affected by the formation of an actin ring involved in creating a tightly enclosed space, known as a resorption lacuna (Novack and Teitelbaum 2008). Switch-associated protein 70 (SWAP-70) deficiency leads to an osteopetrotic phenotype caused by ineffective bone resorption owing to defects in actin ring formation (Roscher et al. 2016). The formation of actin-based sealing rings⁣ is impaired in cortactin-depleted osteoclasts, which exhibit a loss of bone-resorbing function (Tehrani et al. 2006). The presence of (-)-tubaic acid attenuated bone resorption (Figure 4(a)), which correlated with reduced actin ring formation (Figure 3(a)). It inhibited the formation of multinucleated osteoclasts (Figure 1(c)). Osteoclast differentiation and bone resorptive function mainly rely on RANKL-RANK signaling, which activates the MAPK pathways (Fuller et al. 1998). Various studies using a pharmacological inhibitor of MEK-ERK or genetic disruption of *Erk1* have demonstrated the importance of the ERK pathway in osteoclast formation and function (Yan et al. 2007; He et al. 2011). Nakamura et al. observed a significant role of ERK in the survival and polarity of osteoclasts (Nakamura et al. 2003). Additionally, ERK activation by the granulocyte-macrophage colony-stimulating factor (GM-SCF) induces DC-STAMP expression, which promotes cell fusion to generate multinucleated osteoclasts (Lee et al. 2009). Investigation of the molecular mechanism underlying the anti-osteoclastogenic activity of (-)-tubaic acid revealed potent suppression of MEK and ERK phosphorylation (Figure 4(c)). Although the direct target of (-)-tubaic acid has not been clearly identified, impairment of MEK/ERK activation in the presence of (-)-tubaic acid contributes to the attenuation of NFATc1 and subsequent inhibition of osteoclastogenesis. Recently, B591 carrying a 2,3-dihydrobenzofuran core and potently inhibiting the PI3 K/mTOR signaling pathway suppresses feedback activation of ERK and Akt in rhabdomyosarcoma RD and breast cancer cells, indicating that B591 might have enhanced anticancer activity (Zhou et al. 2019). Both ERK and Akt pathways are affected by B591; however, (-)-tubaic acid only suppressed MEK/ERK phosphorylation, which may be attributed to structural differences between the compounds and different cell types.

Collectively, we demonstrated that (-)-tubaic acid exhibits anti-osteoclastogenic and anti-resorptive properties *in vitro*. Furthermore, it attenuates RANKL-mediated activation of the MEK/ERK signaling pathway and down-regulates osteoclast marker expression. However, additional research is required to confirm the *in vivo* efficacy of (-)-tubaic acid in the treatment of osteoclast-related bone diseases. Nonetheless, these results indicate that it has potential applications in the treatment and prevention of bone diseases associated with excessive osteoclast formation and function.

## Data Availability

The data underlying this article are available in the article.
